# Gene‐specific ctDNA dynamics predict tumour burden and survival outcomes in ESCC: A prospective cohort study

**DOI:** 10.1002/ctm2.70446

**Published:** 2025-08-15

**Authors:** Rentong Gu, Tao Liu, Wen Cheng, Mengxing Li, Xiaowei Wang, Hai Jin

**Affiliations:** ^1^ Department of Thoracic Surgery Changhai Hospital Naval Medical University Shanghai China; ^2^ Department of Thoracic Surgery Eastern Hepatobiliary Surgery Hospital Naval Medical University Shanghai China; ^3^ Department of Thoracic Surgery Peking University First Hospital Beijing China; ^4^ Department of Thoracic Surgery Shanghai Pulmonary Hospital School of Medicine Tongji University Shanghai China

**Keywords:** circulating tumour DNA (ctDNA), oesophageal squamous cell carcinoma (ESCC), PIK3CA, prognosis, PTEN, TP53

## Abstract

**Background:**

Oesophageal squamous cell carcinoma (ESCC) remains a highly aggressive malignancy with limited biomarkers for monitoring tumour burden and prognosis. Circulating tumour DNA (ctDNA) has emerged as a promising tool for real‐time disease assessment, but its clinical utility in ESCC remains underexplored.

**Methods:**

In this prospective cohort study, we analysed preoperative and postoperative ctDNA from 54 treatment‐naïve ESCC patients undergoing radical surgery using a 61‐gene panel. Associations between ctDNA mutations, clinicopathological characteristics and survival outcomes were evaluated.

**Results:**

Preoperative ctDNA mutations were detected in 96.3% of patients (52/54), with Tumour Protein 53 (TP53) (59.3%), Phosphatidylinositol‐4,5‐bisphosphate 3‐kinase catalytic subunit alpha (PIK3CA) (31.5%) and Phosphatase and Tensin Homologue (PTEN) (13.0%) being most prevalent. Surgical resection significantly reduced ctDNA positivity (*p* < .0001). Advanced‐stage tumours exhibited higher frequencies of PIK3CA (47.8% vs. 19.4%, *p* = .026) and PTEN mutations (26.1% vs. 3.2%, *p* = .034). Survival analysis revealed that postoperative TP53 ctDNA positivity predicted worse disease‐free survival (DFS; Hazard Ratio (HR) = 3.64, *p *= .005) and overall survival (OS; HR = 3.29, *p* = .009), while PIK3CA positivity was associated with improved OS (*p* = .032). Strikingly, preoperative PTEN ctDNA‐positive patients showed dramatically worse outcomes, with median DFS of 4.01 versus 33.27 months (HR = 7.53, *p* < .001) and OS of 11.80 versus 45.17 months (HR = 5.35, *p* < .001). In multivariate analysis, preoperative PTEN positivity remained the strongest independent prognostic factor for both DFS (HR = 7.28, *p* = .002) and OS (HR = 3.76, *p* = .028), surpassing Tumour, Node, Metastasis (TNM) stage.

**Conclusions:**

Our findings highlight the dynamic role of ctDNA in reflecting ESCC tumour burden and prognosis. While tumour‐agnostic ctDNA analysis showed limited clinical utility, gene‐specific mutations (TP53, PIK3CA and PTEN) demonstrated significant prognostic value. Preoperative PTEN ctDNA positivity emerged as a robust predictor of aggressive disease, suggesting its potential for risk stratification and personalised therapeutic strategies in ESCC.

## Graphical abstract

Notably, *PTEN* mutations in preoperative ctDNA were strongly associated with poor survival, outperforming TNM stage as an independent prognostic marker. Postoperative *TP53* positivity predicted worse outcomes, while *PIK3CA* mutations correlated with improved OS. These findings highlight the potential of gene‐specific ctDNA analysis for risk stratification and personalised treatment in ESCC.

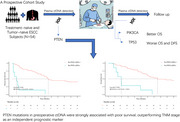

## 1 INTRODUCTION

Oesophageal cancer ranks among the most prevalent and lethal malignancies worldwide.^1^ China exhibits a particularly high incidence, with oesophageal squamous cell carcinoma (ESCC) accounting for approximately 90% of cases, in contrast to Western countries.^2^ While surgery remains the primary recommended treatment, it is associated with substantial perioperative complications and mortality.^3^ Furthermore, the anatomical alterations from digestive tract reconstruction often lead to persistent gastrointestinal symptoms, with 38.9% of 961 postoperative patients reporting at least one such symptom that significantly impaired quality of life.^4^

The SANO trial^5^ demonstrated that active surveillance yielded non‐inferior 2‐year overall survival (OS), compared to standard surgery in patients achieving complete clinical response after neoadjuvant chemoradiotherapy, suggesting potential applicability of a ‘watch‐and‐wait’ strategy. However, 48% of the surveillance group experienced isolated local recurrence. Current post‐diagnosis monitoring relies primarily on imaging, which suffers from limited specificity/sensitivity and radiation exposure, with no standardised approach for lymph node metastasis detection.

Circulating tumour DNA (ctDNA), carrying tumour‐specific genetic alterations with a short half‐life (15 min, 2 h),^6^, ^7^ enables real‐time tumour burden monitoring.^8^ While next‐generation sequencing (NGS)‐based ctDNA analysis^9^ has proven valuable for cancer screening, treatment response assessment and prognosis prediction in various malignancies,^10^, ^11^, ^12^, ^13^, ^14^ its application in ESCC remains underdeveloped.

This study aimed to determine the value of mutant ctDNA as a dynamic biomarker for the real‐time assessment of tumour burden and prognostic stratification in patients with ESCC.

## 2 MATERIALS AND METHODS

### 2.1 Patients

This prospective cohort study enrolled 54 patients with histologically confirmed ESCC who underwent radical surgical resection at the Department of Thoracic Surgery between August 2015 and December 2019. All patients underwent intraoperative frozen‐section examination to confirm negative upper resection margins, with ≥5 cm margins maintained for mid/lower thoracic ESCC and histologically confirmed negative margins for upper thoracic tumours despite anatomical constraints. The study population was selected based on stringent inclusion criteria: (1) pathologically confirmed ESCC diagnosis; (2) complete surgical resection with curative intent; (3) treatment‐naïve status at the time of initial blood sampling (no prior chemotherapy, radiotherapy or targeted therapy); and (4) no history of other malignancies. The study protocol was approved by the Institutional Review Board in accordance with the Declaration of Helsinki, and all participants provided written informed consent prior to study enrollment.

Comprehensive baseline clinicopathological characteristics were prospectively collected for all study participants, including demographic data (age, gender), tumour staging (American Joint Committee on Cancer/Union for International Cancer Control (AJCC/UICC) 8th edition TNM classification), histological differentiation grade and pretreatment serum tumour marker profiles. To ensure standardised disease monitoring, we implemented a rigorous follow‐up protocol: quarterly clinical assessments (physical examination, contrast‐enhanced CT imaging and serum biomarker analysis) during the initial 2‐year post‐treatment period, transitioning to biannual evaluations thereafter. The median follow‐up duration was 36 months (range: 12–60 months), with disease progression objectively determined using RECIST 1.1 criteria.

### 2.2 ctDNA analysis

Peripheral blood samples (EDTA tubes) were processed within 3 h of collection (4°C) and subjected to rigorous quality control: cfDNA was quantified using Qubit 3.0 Fluorometer and assessed via Agilent 2100 Bioanalyzer, requiring (1) characteristic 160 bp cfDNA peak without gDNA contamination and (2) minimum 10 ng total cfDNA yield for inclusion. Plasma cell‐free DNA (cfDNA) and matched leukocyte genomic DNA (gDNA) were subjected to targeted NGS using a 61‐gene panel (Table S1). Sequencing was performed on Illumina HiSeq 2500 platforms (Illumina) at Shanghai Yunsheng Medical Laboratory, employing the Accu‐Act panel with Firefly NGS technology (Yunsheng Medical Laboratory) for cfDNA analysis. Our hybrid capture‐based sequencing achieved a median depth of 9275X, enabling detection of somatic variants at ≥.02% variant allele frequency (95% confidence). Proprietary bioinformatics pipelines incorporating unique molecular identifiers and dual filtering strategies: (1) exclusion of all leukocyte‐detected variants (VAF = 0) and (2) COSMIC database cross‐referencing to remove CHIP‐associated hotspots were used for variant calling, with germline variants and clonal hematopoietic mutations systematically excluded from final analysis. Fragment size profiles confirmed ctDNA enrichment through the characteristic 160 bp peak.

### 2.3 Statistical analyses

Categorical variables were expressed as frequencies (percentages), while continuous variables were presented as mean ± SD (normally distributed) or median with Interquartile Range (IQR) (non‐normally distributed). Group comparisons employed independent *t*‐tests (normal distributions with homogeneous variance) or Mann–Whitney U tests (non‐normal distributions) for continuous variables; χ^2^ tests or Fisher's exact tests for categorical variables; and McNemar's tests for paired categorical data. Survival analyses utilised Kaplan–Meier methodology (log‐rank test) with Cox proportional hazards regression for multivariate adjustment. All statistical analyses and visualisations were performed using GraphPad Prism 9 (GraphPad Software) and R 4.3.1 (R Foundation for Statistical Computing), with two‐sided *p*‐values < .05 considered statistically significant.

## 3 RESULTS

### 3.1 Clinical and pathological characteristics of the study cohort

The clinicopathological characteristics of the 54 ESCC patients are summarised in Table S2. The cohort had a median age of 65.0 years (IQR 60.3–70.0) at diagnosis and median Body Mass Index (BMI) of 23.4 kg/m^2^ (IQR 21.9–24.9). Male predominance was observed (81.5%, *n* = 44) versus females (18.5%, *n* = 10). Regarding lifestyle factors, 40.7% had combined smoking and drinking history, while others had smoking only (14.8%), drinking only (5.6%) or neither habit (38.9%).

Most tumours originated in the middle thoracic oesophagus (68.5%), and Ivor Lewis oesophagectomy was performed in 79.6% of cases. All patients underwent intraoperative frozen‐section examination to confirm negative upper resection margins to ensure R0 resection. For mid/lower thoracic ESCC, we maintained a ≥5 cm upper resection margin, while for upper thoracic tumours (requiring cervical anastomosis in McKeown procedures), we ensured histologically negative margins despite anatomical constraints. All patients received standardised two‐field (thoracoabdominal) lymphadenectomy.

Pathological examination revealed median tumour diameter of 4.0 cm (IQR 3.0–4.5), with moderately differentiated histology predominating (68.5%). Angiolymphatic invasion was present in 25.9% and neurological invasion in 22.2%. According to the 8th AJCC/UICC staging system, cases were distributed as follows: Stage I (25.9%, *n* = 14), Stage II (29.6%, *n* = 16), Stage III (40.7%, *n *= 22) and Stage IV (3.7%, *n* = 2). Adjuvant therapy was administered to 37.0% (*n* = 20) of patients postoperatively (standard chemoradiotherapy: *n *= 13; radiotherapy alone: *n* = 3; precautionary radiotherapy for narrow margins: *n* = 4). Among 26 guideline‐eligible cases, five patients declined treatment and five had undocumented regimens.

### 3.2 Circulating tumour DNA mutation profiling

**FIGURE 1:**
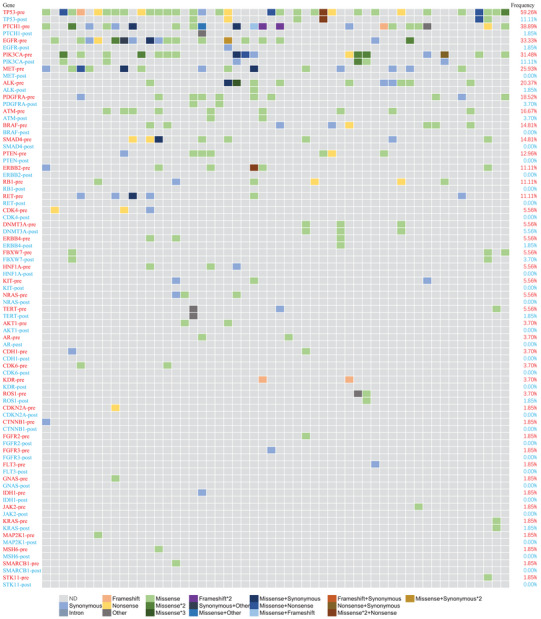
Circulating tumour DNA (ctDNA) somatic mutations pre/postoperative in 54 oesophageal squamous cell carcinoma (ESCC) patients.

Using a 61‐gene panel, we performed comprehensive genomic analysis of plasma cfDNA (preoperative and postoperative Day 7) and matched leukocyte gDNA in all 54 patients (Figure 1). Multiple somatic mutations were detected in 96.3% (52/54) of preoperative ctDNA samples. TP53 was the most frequently mutated gene (59.3% prevalence), followed by 13 additional genes with mutation frequencies >9.3% (≥ 5 cases): PTCH1, EGFR, PIK3CA, MET, ALK, PDGFRA, ATM, BRAF, SMAD4, PTEN, ERBB3, RB1 and RET. Missense mutations represented the predominant variant type.

In postoperative ctDNA analysis, TP53 and PIK3CA remained the most prevalent alterations (11.1% each, 6/54), while all other genes showed mutation frequencies ≤3.7% (2/54).

### 3.3 Mutation analysis associated with tumour burden

#### 3.3.1 Surgical impact on ctDNA mutation status

**FIGURE 2:**
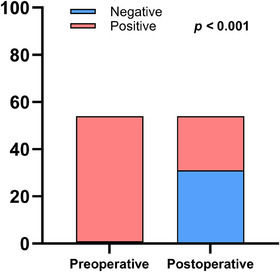
Surgical impact on ctDNA status in ESCC.

Using the tumour‐agnostic strategy, McNemar's test with continuity correction revealed a statistically significant reduction in ctDNA positivity following surgical resection (χ^2^ = 28.03, df = 1, *p* < .0001), with 55.6% (30/54) of patients converting from preoperative positivity to postoperative negativity (Figure 2).

#### 3.3.2 Association between preoperative gene‐specific ctDNA and TNM staging

**FIGURE 3:**
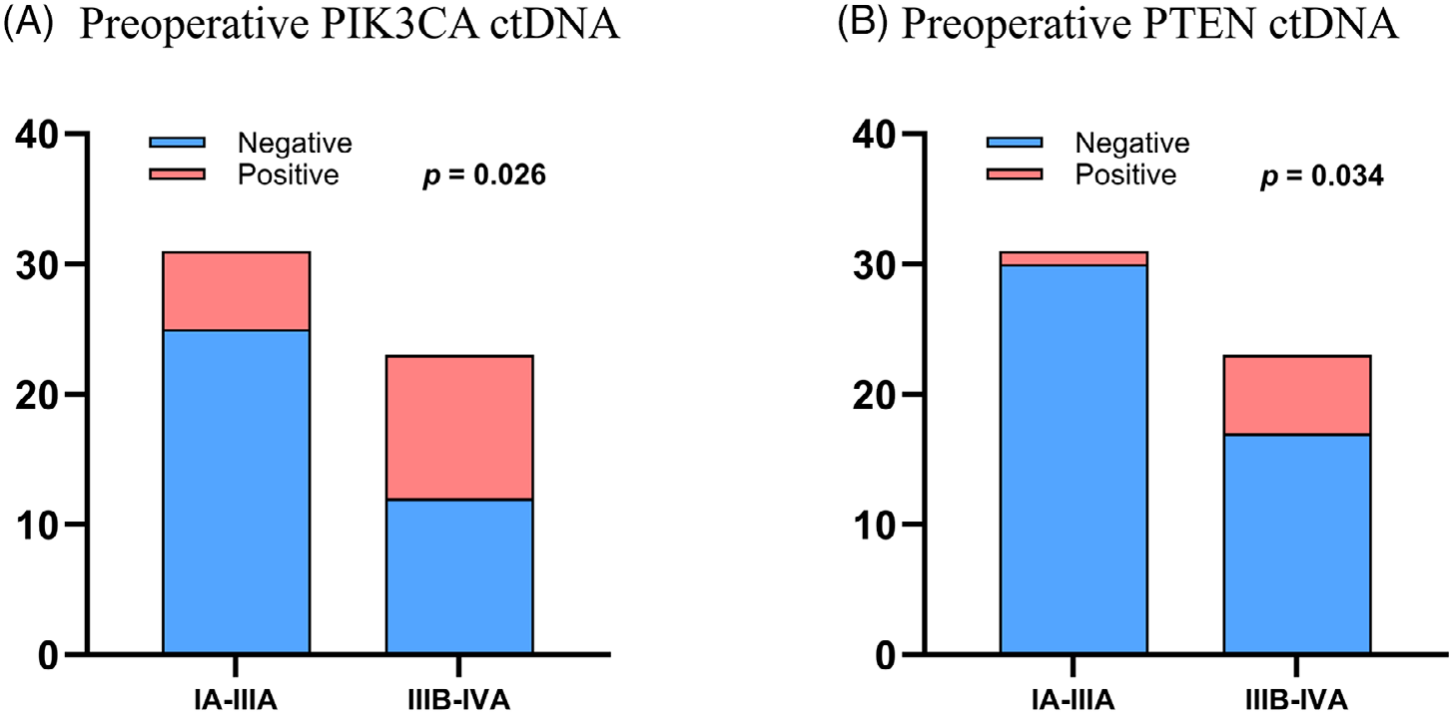
Preoperative different gene‐specific ctDNA associations with tumour burden. (A) Association between preoperative PIK3CA ctDNA and tumour burden. (B) Association between preoperative PTEN ctDNA and tumour burden. ctDNA, circulating tumour DNA.

The cohort was stratified by TNM stage into two comparative groups: Stage IA–IIIA (*n* = 31) representing regional disease and Stage IIIB–IVA (*n* = 23) representing advanced disease. We performed individual analyses for all 14 genes with preoperative ctDNA detection in ≥5 cases using χ^2^ or Fisher's exact tests as appropriate. The χ^2^ analysis showed PIK3CA mutations were significantly more frequent in the advanced stage group (47.8%, 11/23) than the regional stage group (19.4%, 6/31; χ^2^ = 4.96, *p* = .026), with an overall mutation prevalence of 31.5% (17/54). PTEN mutations differed significantly between groups (Fisher's exact *p* = .034), occurring in 26.1% (6/23) of the advanced stage group versus 3.2% (1/31) of the regional stage group, with an overall frequency of 13.0% (7/54). Only PIK3CA and PTEN mutations showed statistically significant differences in prevalence between groups (Figure 3). The remaining 12 genes (TP53, PTCH1, EGFR, MET, ALK, PDGFRA, ATM, BRAF, SMAD4, ERBB3, RB1 and RET) demonstrated no significant associations (all *p* > .05; see Table S3 for complete results).

### 3.4 Prognostic outcomes and survival analysis

All 54 enrolled patients completed the 5‐year follow‐up period, demonstrating a 64.8% (35/54) recurrence rate with a median disease‐free survival (DFS) of 31.04 months (95% CI: 23.10–NA) and a 61.1% (33/54) mortality rate with median OS of 39.42 months (95% CI: 30.54–NA).

Using the tumour‐agnostic strategy, we stratified 54 patients with completed 5‐year follow‐up based on the presence or absence of Class I/II gene variants in postoperative plasma ctDNA. Survival analysis showed no significant differences in 5‐year OS or DFS between the two groups (Figure S1).

Further stratification by postoperative TP53 ctDNA status revealed significantly shorter median DFS in the TP53 ctDNA‐positive group (4.82 vs. 32.50 months; *p* = .003, HR = 3.643, 95% CI 1.480–8.970) and inferior median OS (19.08 vs. 43.43 months; *p* = 0.006, HR = 3.287, 95% CI 1.341–8.056).

For PIK3CA variants, while no significant DFS difference was observed (median 51.48 vs. 29.57 months; *p* = .266), PIK3CA ctDNA‐positive cases exhibited prolonged OS (not reached vs. 35.93 months; *p* = .032, HR = 0.151, 95% CI .021–1.110).

**FIGURE 4:**
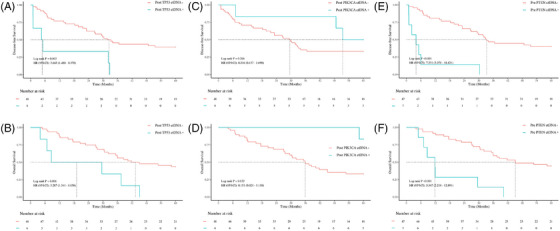
Prognostic impact of postoperative gene‐specific ctDNA status in ESCC patients. (A) Postoperative TP53 ctDNA status (positive vs. negative) and disease‐free survival (DFS). (B) Postoperative TP53 ctDNA status (positive vs. negative) and overall survival (OS). (C) Postoperative PIK3CA ctDNA status (positive vs. negative) and DFS. (D) Postoperative PIK3CA ctDNA status (positive vs. negative) and OS. (E) Preoperative PTEN ctDNA status (positive vs negative) and DFS. (F) Preoperative PTEN ctDNA status (positive vs negative) and OS. All comparisons were performed using log‐rank tests.

Since no PTEN mutations were detected postoperatively, we evaluated preoperative PTEN ctDNA status. PTEN ctDNA‐positive patients had markedly reduced median DFS (4.01 vs. 33.27 months; *p* < .001, HR = 7.531, 95% CI 3.078–18.421) and OS (11.80 vs. 45.17 months; *p* < .001, HR = 5.347, 95% CI 2.218–12.891; Figure 4).

**FIGURE 5:**
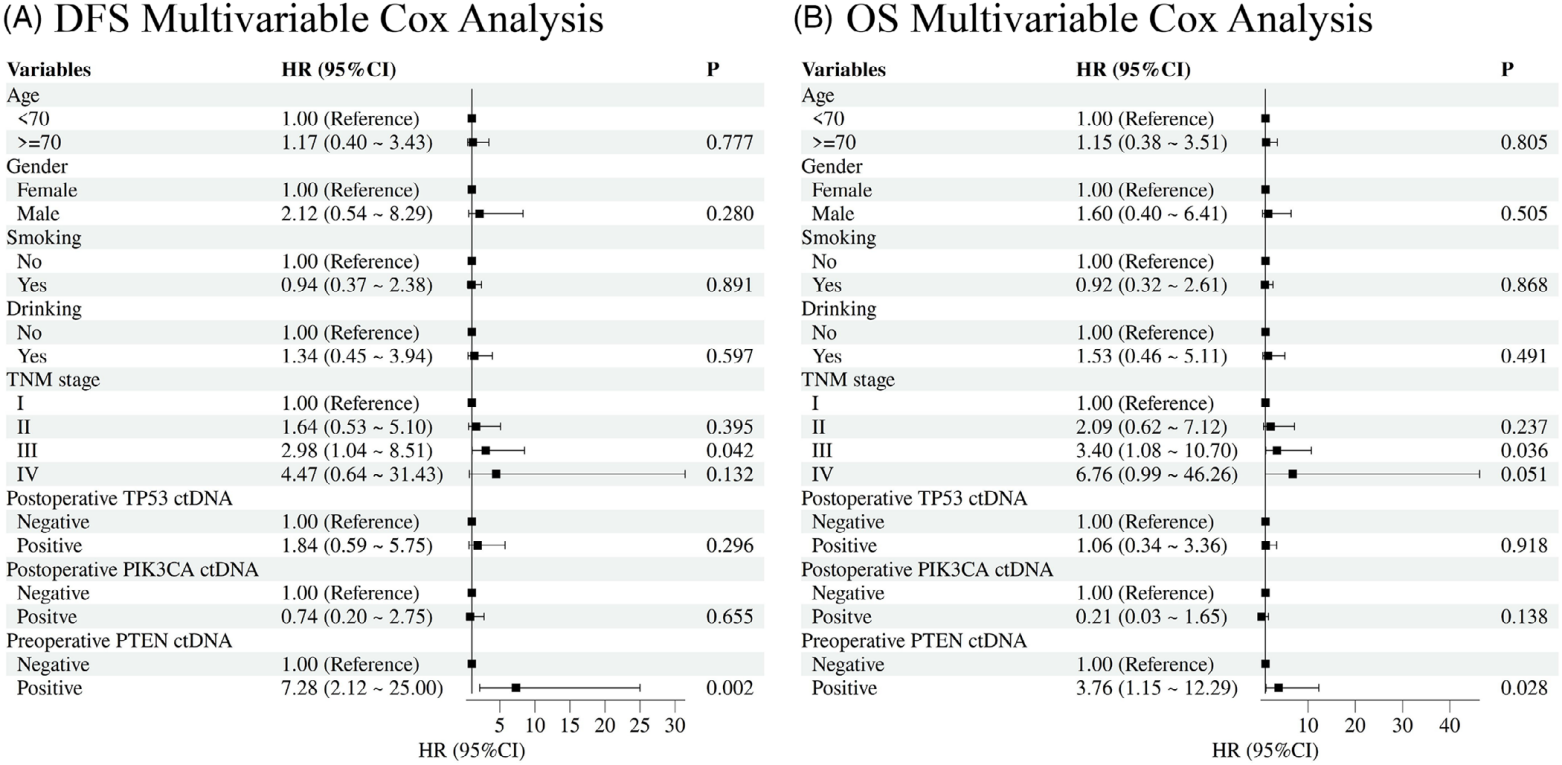
Independent prognostic factors for survival in multivariable Cox analysis. (A) DFS predictors: preoperative PTEN ctDNA (HR = 7.28, *p* = .002) and TNM Stage III (HR = 2.98, *p* = .042). (B) OS predictors: preoperative PTEN ctDNA (HR = 3.76, *p* = .028) and TNM Stage III (HR = 3.40, *p* = .036).

Cox regression analyses were performed to evaluate the impact of clinical and molecular variables on DFS and OS. In univariate analysis for DFS, TNM Stage III (HR = 4.40, 95%CI 1.64–11.83, *p* = .003), postoperative TP53 ctDNA positivity (HR = 3.64, 1.48–8.97, *p* = .005), and preoperative PTEN ctDNA positivity (HR = 7.53, 3.08–18.42, *p* < .001) emerged as significant risk factors (Figure S2A). Multivariate analysis confirmed preoperative PTEN ctDNA positivity (HR = 7.28, 2.12–25.00, *p* = 0‐.002) and TNM Stage III (HR = 2.98, 1.04–8.51, *p* = .042) as independent predictors of worse DFS (Figure 5A).

For OS, univariate analysis identified TNM Stage III/IV (HR = 4.92, 95%CI 1.66–14.54, *p* = .004; HR = 5.73, 1.03–31.99, *p* = .046), postoperative TP53 ctDNA positivity (HR = 3.29, 1.34–8.06, *p* = .009), preoperative PTEN ctDNA positivity (HR = 5.35, 2.22–12.89, *p* < .001), and alcohol consumption (HR = 2.13, 1.06–4.26, *p* = .033) as significant adverse factors, while postoperative PIK3CA ctDNA positivity showed a protective trend (HR = 0.15, 0.02–1.11, *p* = .063; Figure S2B). In multivariate analysis, only preoperative PTEN ctDNA positivity (HR = 3.76, 1.15–12.29, *p* = .028) and TNM Stage III (HR = 3.40, 1.08–10.70, *p* = .036) remained independently significant (Figure 5B).

## 4 DISCUSSION

This is the first prospective cohort study to conduct separate analyses of different gene mutations in ctDNA‐positive ESCC. We performed tumour‐agnostic ctDNA analysis using a 61‐gene panel (Class I/II cancer‐related genes) on preoperative and postoperative plasma samples from 54 treatment‐naïve ESCC patients. Following germline mutation filtering, TP53 emerged as the most prevalent mutation in both preoperative and postoperative ctDNA. Moreover, multiple mutations were identified in most patients' preoperative ctDNA, reflecting the heterogeneity of ESCC.^15^

First, we analysed the relationship between preoperative ctDNA status and tumour burden. Current ctDNA detection strategies can be divided into tumour‐informed and tumour‐agnostic approaches based on whether tumour tissue mutations are referenced. Multiple studies^16^, ^17^ have confirmed that tumour‐informed strategy is significantly more accurate than tumour‐agnostic one. However, tumour‐informed strategy requires individualised customisation, is costly and is limited by tumour heterogeneity and the impact of post‐treatment tumour subclonal mutations. In contrast, tumour‐agnostic strategy offers higher sensitivity but lower specificity, with established clinical value currently limited to lung,^18^, ^19^ breast^20^ and colorectal cancers.^21^ We speculate this is related to the presence of high‐frequency driver gene mutations in these cancer populations. In our study, 98.15% (53/54) of patients were preoperative ctDNA‐positive. Additionally, we detected ctDNA positivity in three Stage 0 ESCC patients who were excluded from this study, further validating the low specificity of tumour‐agnostic ctDNA detection and its inability to stratify populations with different tumour burdens. However, we observed that 55.56% of patients became ctDNA‐negative postoperatively, demonstrating ctDNA's ability to reflect tumour burden in real time with high sensitivity. Therefore, we conducted separate analyses of different genes in preoperative ctDNA‐positive cases to identify specific ctDNA mutations of tumour burden. We found that preoperative PIK3CA ctDNA and PTEN ctDNA positivity could distinguish patients with locally advanced ESCC. Some breast cancer studies have also shown that PIK3CA mutations in tumour tissue^22^, ^23^ can reflect tumour burden. A prostate cancer study^24^ found that patients with PTEN mutations in tumour tissue had higher Positron Emission Tomography‐Computed Tomography (PET‐CT) positivity rates in lesions, indirectly indicating differences in tumour burden.

Second, we analysed the relationship between ctDNA status and survival outcomes. We found that postoperative ctDNA positivity under the tumour‐agnostic strategy did not indicate worse 5‐year OS or DFS. However, when analysing TP53 ctDNA separately, patients with postoperative TP53 ctDNA positivity had significantly worse 5‐year OS and DFS than negative patients, a finding consistent with Zhang et al.’s study in advanced breast cancer.^25^ Interestingly, patients with postoperative PIK3CA ctDNA positivity showed significantly better 5‐year OS than negative patients, similar to results obtained by Hou et al. in their tumour tissue analysis of 96 ESCC patients.^26^ Notably, while preoperative PIK3CA positivity indicated more advanced tumour staging, postoperative PIK3CA positivity predicted better prognosis. This paradoxical observation has also been reported in breast cancer studies of PIK3CA, where some studies found that in early‐stage breast cancer, PIK3CA kinase domain mutations reduced cell chemotaxis, thereby decreasing cancer cell migration and metastasis and improving prognosis.^27^, ^28^ In contrast, other studies^29^ showed that PIK3CA mutations led to worse outcomes in advanced breast cancer patients not receiving targeted therapy. Kaplan–Meier analysis revealed significantly inferior survival in preoperative PTEN ctDNA‐positive patients versus negative counterparts, aligning with prior breast cancer findings.^30^ Multivariate Cox regression adjusting for sex, age and tumour stage confirmed preoperative PTEN ctDNA positivity as an independent prognostic factor for both 5‐year OS and DFS.

In this study, the low mutation frequencies of PIK3CA, PTEN and postoperative TP53—genes associated with tumour burden and prognosis—further reflect ESCC's heterogeneity and confirm the absence of high‐frequency driver genes in this malignancy. Notably, PTEN mutations demonstrated clinically meaningful effect sizes (HR > 5) with > 89% statistical power, whereas TP53 showed significance but suboptimal power (57.5%–67%), and PIK3CA analysis was limited by a single event. In our multivariable Cox analysis, we included TP53, PIK3CA and PTEN mutations along with key clinical variables to explore their potential prognostic value. While the model's statistical power was limited by the cohort size, these specific genes were selected based on their biological plausibility in ESCC pathogenesis. Therefore, we conclude that tumour‐agnostic large‐panel ctDNA testing throughout the disease course has limited clinical value for monitoring ESCC. However, small‐panel ctDNA testing incorporating these genes, particularly PTEN, could be valuable for identifying specific patient subgroups, such as those with rapidly progressive, poor‐prognosis ESCC resembling small cell lung cancer, potentially leading to fundamental changes in treatment strategies.

This study has several limitations. First, the small sample size necessitates larger studies to validate ctDNA's efficacy. Second, one preoperative ctDNA‐negative case (pT2N1M0, Stage IIIA) subsequently developed recurrence and died within 5 years, indicating potential limitations in our 61‐gene panel's coverage. The panel's modest size (61 genes) unavoidably excluded certain ESCC drivers (NOTCH1, NFE2L2), which may explain this false‐negative result. Separately, the detection of relatively high frequencies of typically rare mutations (ALK 20.37%, RET 11.11%) likely reflects our small cohort size (*n *= 54) rather than true biological prevalence. Additionally, while our bioinformatics pipeline excluded known CHIP‐associated variants, ultra‐low‐frequency CHIP mutations below detection thresholds may persist, which could potentially influence the results at marginal VAF levels. Third, in the Kaplan–Meier analysis of OS for postoperative PIK3CA ctDNA‐positive patients, the limited number of events (*n* = 1) in the positive group resulted in wide confidence intervals for the hazard ratio, potentially compromising the reliability of this finding. Fourth, our single timepoint (Day 7) postoperative sampling, while supported by recent evidence demonstrating nearly identical ctDNA positivity rates between early (0–2 weeks) and later (2–8 weeks) postoperative periods,^31^ may not fully distinguish true MRD from perioperative artifacts. Fifth, as most follow‐ups occurred at local institutions, only sporadic blood samples were available at non‐standardised timepoints, precluding systematic recurrence‐phase detection. For patients receiving postoperative adjuvant therapy, this also prevented continuous ctDNA monitoring during treatment. This decentralized follow‐up model, though reflective of real‐world practice in China's tiered healthcare system, limited our ability to implement multi‐timepoint sampling protocols. Furthermore, while lymph node extranodal extension (ENE) is an established poor prognostic factor in ESCC,^32^ standardised evaluation of ENE in lymph node metastases was not routinely performed during the study period, which may represent a potential confounding factor in survival analysis.

As an exploratory study, we are currently expanding the cohort to validate these findings, with particular attention to stage‐specific and molecularly stratified ESCC populations (including neoadjuvant‐treated patients), mutation‐specific statistical power and clinical effect sizes, comparative performance of ctDNA versus conventional biomarkers (imaging/pathological staging) and implementation of standardised multi‐institutional sampling protocols to address longitudinal monitoring challenges, with future analyses incorporating serial ctDNA measurements alongside tissue biomarker panels.

## 5 CONCLUSION

Surgical resection effectively reduced ctDNA detection, with over half of the patients clearing circulating tumour DNA postoperatively. Advanced‐stage tumours showed higher frequencies of preoperative ctDNA‐positive PIK3CA and PTEN mutations, suggesting their role in disease progression. The persistence of TP53 ctDNA postoperatively identified patients with substantially increased risk of poor survival, while PIK3CA ctDNA positivity showed potential protective effects. Most significantly, preoperative PTEN ctDNA positivity emerged as the strongest independent predictor of poor outcomes.

## AUTHOR CONTRIBUTIONS

**Rentong Gu**: Conceptualisation; investigation; formal analysis; writing—original draft. **Tao Liu, Wen Cheng, Mengxing Li**: Investigation; data curation; writing—original draft. **Xiaowei Wang**: Methodology; supervision; writing—review and editing. **Hai Jin**: Conceptualisation; methodology; funding acquisition; supervision; writing—review and editing.

## CONFLICT OF INTEREST STATEMENT

The authors declare no conflicts of interest.

## ETHICS STATEMENT

This study was approved by the Institutional Review Board (IRB) (Approval No. CHEC2020‐021). All participants provided written informed consent after receiving a detailed explanation of the study purpose, procedures, risks, benefits and their rights. The research was conducted in accordance with the Declaration of Helsinki and relevant ethical guidelines.

## Supporting information



Supporting Information

Supporting Information

Supporting Information

Supporting Information

Supporting Information

## Data Availability

The raw sequencing data generated in this study have been deposited in the China National Genebank (CNGB, https://db.cngb.org/cnsa/) under accession number CNP0001778. Non‐sequencing raw data (including clinical records and imaging files) are subject to access restrictions in accordance with the Administrative Regulations of the People's Republic of China on Human Genetic Resources (http://www.gov.cn/zhengce/content/2019‐06/10/content_5398829.htm). Processed analytical data (anonymized results) are available from the corresponding author upon reasonable request. Researchers may apply for access to restricted datasets by submit‐ting a formal request to CNGBdb@cngb.org, specifying: (1) the study accession code (CNP0001778), (2) detailed data requirements, and (3) intended research purpose.

## References

[1] Sung H , Ferlay J , Siegel RL , et al. Global cancer statistics 2020: GLOBOCAN estimates of incidence and mortality worldwide for 36 cancers in 185 countries. CA Cancer J Clin. 2021;71(3):209‐249. doi:10.3322/caac.21660 33538338

[2] Chen W , Zheng R , Baade PD , et al. Cancer statistics in China, 2015. CA Cancer J Clin. 2016;66(2):115‐132. doi:10.3322/caac.21338 26808342

[3] Low DE , Kuppusamy MK , Alderson D , et al. Benchmarking complications associated with esophagectomy. Ann Surg. 2019;269(2):291‐298. doi:10.1097/SLA.0000000000002611 29206677

[4] van Erning FN , Nieuwenhuijzen GAP , van Laarhoven HWM , et al. Gastrointestinal symptoms after resection of esophagogastric cancer: a longitudinal study on their incidence and impact on patient‐reported outcomes. Ann Surg Oncol. 2023;30(13):8203‐8215. doi:10.1245/s10434-023-13952-z 37523120

[5] Van der Wilk BJ , Eyck BM , Wijnhoven BPL , et al. Neoadjuvant chemoradiotherapy followed by surgery versus active surveillance for oesophageal cancer (SANO‐trial): a phase‐III stepped‐wedge cluster randomised trial. Paper presented at: Congress of the European‐Society‐for‐Medical‐Oncology (ESMO); October 20–24, 2023; Madrid, Spain.

[6] Ye Q , Ling S , Zheng S , Xu X . Liquid biopsy in hepatocellular carcinoma: circulating tumor cells and circulating tumor DNA. Mol Cancer. 2019;18(1):114. doi:10.1186/s12943-019-1043-x 31269959 PMC6607541

[7] Egyud M , Tejani M , Pennathur A , et al. Detection of circulating tumor DNA in plasma: a potential biomarker for esophageal adenocarcinoma. Ann Thorac Surg. 2019;108(2):343‐349. doi:10.1016/j.athoracsur.2019.04.004 31059681 PMC6676214

[8] Azad TD , Chaudhuri AA , Fang P , et al. Circulating tumor DNA analysis for detection of minimal residual disease after chemoradiotherapy for localized esophageal cancer. Gastroenterology. 2020;158(3):494‐505. doi:10.1053/j.gastro.2019.10.039 31711920 PMC7010551

[9] Haber DA , Velculescu VE . Blood‐based analyses of cancer: circulating tumor cells and circulating tumor DNA. Cancer Discov. 2014;4(6):650‐661. doi:10.1158/2159-8290.CD-13-1014 24801577 PMC4433544

[10] Pantel K , Alix‐Panabières C . Liquid biopsy and minimal residual disease—latest advances and implications for cure. Nat Rev Clin Oncol. 2019;16(7):409‐424. doi:10.1038/s41571-019-0187-3 30796368

[11] Chen X , Gole J , Gore A , et al. Non‐invasive early detection of cancer four years before conventional diagnosis using a blood test. Nat Commun. 2020;11(1):3475. doi:10.1038/s41467-020-17316-z 32694610 PMC7374162

[12] Abbosh C , Birkbak NJ , Wilson GA , et al. Corrigendum: phylogenetic ctDNA analysis depicts early‐stage lung cancer evolution. Nature. 2018;554(7691):264. doi:10.1038/nature25161 29258292

[13] Duffy MJ , McDermott EW , Crown J . Blood‐based biomarkers in breast cancer: from proteins to circulating tumor cells to circulating tumor DNA. Tumour Biol. 2018;40(5):1010428318776169. doi:10.1177/1010428318776169 29775157

[14] Wang Y , Yang L , Bao H , et al. Utility of ctDNA in predicting response to neoadjuvant chemoradiotherapy and prognosis assessment in locally advanced rectal cancer: a prospective cohort study. PLoS Med. 2021;18(8):e1003741. doi:10.1371/journal.pmed.1003741 34464382 PMC8407540

[15] Hao JJ , Lin DC , Dinh HQ , et al. Spatial intratumoral heterogeneity and temporal clonal evolution in esophageal squamous cell carcinoma. Nat Genet. 2016;48(12):1500‐1507. doi:10.1038/ng.3683 27749841 PMC5127772

[16] Chen K , Yang F , Shen H , et al. Individualized tumor‐informed circulating tumor DNA analysis for postoperative monitoring of non‐small cell lung cancer. Cancer Cell. 2023;41(10):1749‐1762.e6. doi:10.1016/j.ccell.2023.08.010 37683638

[17] Moding EJ , Nabet BY , Alizadeh AA , Diehn M , Detecting liquid remnants of solid tumors: circulating tumor DNA minimal residual disease. Cancer Discov. 2021;11(12):2968‐2986. doi:10.1158/2159-8290.CD-21-0634 34785539 PMC8976700

[18] Abbosh C , Birkbak NJ , Wilson GA , et al. Phylogenetic ctDNA analysis depicts early‐stage lung cancer evolution [published correction appears in Nature. 2018;554(7691):264]. Nature. 2017;545(7655):446‐451. doi:10.1038/nature22364 28445469 PMC5812436

[19] Abbosh C , Frankell AM , Harrison T , et al. Tracking early lung cancer metastatic dissemination in TRACERx using ctDNA. Nature. 2023;616(7957):553‐562. doi:10.1038/s41586-023-05776-4 37055640 PMC7614605

[20] Garcia‐Murillas I , Schiavon G , Weigelt B , et al. Mutation tracking in circulating tumor DNA predicts relapse in early breast cancer. Sci Transl Med. 2015;7(302):302ra133. doi:10.1126/scitranslmed.aab0021 26311728

[21] Tie J , Wang Y , Tomasetti C , et al. Circulating tumor DNA analysis detects minimal residual disease and predicts recurrence in patients with stage II colon cancer. Sci Transl Med. 2016;8(346):346ra92. doi:10.1126/scitranslmed.aaf6219 PMC5346159 27384348

[22] Nakai M , Yamada T , Sekiya K , et al. Use of liquid biopsy to detect PIK3CA mutation in metastatic breast cancer. J Nippon Med Sch. 2022;89(1):66‐71. doi:10.1272/jnms.JNMS.2022_89-107 33692304

[23] Thao DT , Thanh NP , Quyen DV , Khai LT , Song LH , Trung NT . Identification of breast cancer‐associated PIK3CA H1047R mutation in blood circulation using an asymmetric PCR assay. PLoS One. 2024;19(8):e0309209. doi:10.1371/journal.pone.0309209 39197004 PMC11356436

[24] Yining W , Qiaochu C , Liangrong W , Cheng W , Jianjun L . Correlation between PTEN/TP53 expression and molecular imaging phenotypes in primary prostate cancer. Chin J Nucl Med Mol Imaging. 2025;45(05):257‐262.

[25] Zhang L , Sun S , Zhao X , et al. Prognostic value of baseline genetic features and newly identified TP53 mutations in advanced breast cancer. Mol Oncol. 2022;16(20):3689‐3702. doi:10.1002/1878-0261.13297 35971249 PMC9580879

[26] Hou J , Jiang D , Zhang J , et al. Frequency, characterization, and prognostic analysis of PIK3CA gene mutations in Chinese esophageal squamous cell carcinoma. Hum Pathol. 2014;45(2):352‐358. doi:10.1016/j.humpath.2013.09.011 24360885

[27] Pang H , Flinn R , Patsialou A , et al. Differential enhancement of breast cancer cell motility and metastasis by helical and kinase domain mutations of class IA phosphoinositide 3‐kinase. Cancer Res. 2009;69(23):8868‐8876. doi:10.1158/0008-5472.CAN-09-1968 19903845 PMC2793177

[28] Yates LR , Knappskog S , Wedge D , et al. Genomic evolution of breast cancer metastasis and relapse. Cancer Cell. 2017;32(2):169‐184.e7. doi:10.1016/j.ccell.2017.07.005 28810143 PMC5559645

[29] Fillbrunn M , Signorovitch J , André F , et al. PIK3CA mutation status, progression and survival in advanced HR + /HER2‐ breast cancer: a meta‐analysis of published clinical trials. BMC Cancer. 2022;22(1):1002. doi:10.1186/s12885-022-10078-5 36131248 PMC9490901

[30] Li S , Shen Y , Wang M , et al. Loss of PTEN expression in breast cancer: association with clinicopathological characteristics and prognosis. Oncotarget. 2017;8(19):32043‐32054. doi:10.18632/oncotarget.16761 28410191 PMC5458267

[31] Cohen SA , Kasi PM , Aushev VN , et al. Kinetics of postoperative circulating cell‐free DNA and impact on minimal residual disease detection rates in patients with resected stage I‐III colorectal cancer. J Clin Oncol. 2023;41(4 suppl) ASCO GI 2023 abstr 5.

[32] Tu CC , Hsu PK , Chien LI , et al. Prognostic histological factors in patients with esophageal squamous cell carcinoma after preoperative chemoradiation followed by surgery. BMC Cancer. 2017;17(1):62. doi:10.1186/s12885-017-3063-5 28103913 PMC5244588

